# The impact of psychopathology on academic performance in school-age children and adolescents

**DOI:** 10.1038/s41598-022-08242-9

**Published:** 2022-03-11

**Authors:** Mireia Pagerols, Raquel Prat, Cristina Rivas, Gemma Español-Martín, Júlia Puigbó, Èlia Pagespetit, Josep Maria Haro, Josep Antoni Ramos-Quiroga, Miquel Casas, Rosa Bosch

**Affiliations:** 1 SJD MIND Schools Program, Hospital Sant Joan de Déu, Institut de Recerca Sant Joan de Déu, Esplugues de Llobregat, Spain; 2 Centre for Health and Social Care Research (CEES), University of Vic−Central University of Catalonia (UVic−UCC), Vic, Spain; 3 Servei de Psiquiatria, Vall d’Hebron Hospital Universitari, Barcelona, Spain; 4 Departament de Psiquiatria i Medicina Legal, Universitat Autònoma de Barcelona, Bellaterra, Spain; 5 Grup de Psiquiatria, Salut Mental i Addiccions, Vall d’Hebron Institut de Recerca (VHIR), Vall d’Hebron Hospital Universitari, Barcelona, Spain; 6 Research and Developmental Unit, Parc Sanitari Sant Joan de Déu, Sant Boi de Llobregat, Spain; 7 CIBER de Salud Mental (CIBERSAM), Instituto de Salud Carlos III, Madrid, Spain

**Keywords:** Risk factors, Psychiatric disorders, ADHD, Anxiety, Depression, Psychology, Disease prevention, Comorbidities

## Abstract

Psychiatric symptoms have consistently been associated with negative educational outcomes. However, possible confounding variables, such as comorbid mental and environmental conditions, have not been well addressed. This study examined whether mental health problems were significantly linked to academic performance in a Spanish school-based sample, after adjustment for co-occurring psychiatric symptoms and multiple contextual factors. Parents completed a questionnaire regarding child’s sociodemographic characteristics (i.e., gender, age, type of school, socioeconomic status, ethnicity), stressful events (i.e., adoption, parental divorce/separation, grade retention) and lifestyle (i.e., diet, sleep, screen time), along with the Child Behavior Checklist (CBCL). Academic performance was obtained from school records. The sample comprised 7036 students aged 5–17 with full data on the CBCL. Mixed-effects ordinal logistic regression analyses were conducted to investigate the association between psychopathology and academic achievement, controlling for potential confounders. When examined separately, higher scores on the CBCL scales were related to lower grades, regardless of sociodemographic factors. However, after controlling for the presence of other psychiatric symptoms, we found that students who reported more anxious/depressed and thought problems were less likely to perform poorly, while those with increased levels of attention problems and delinquent behavior had higher risk for academic underachievement. These associations remained mainly the same once stressful events and lifestyle were taken into account. This investigation demonstrates that anxious/depressed symptoms, thought problems, attention problems, and delinquent behavior are independently associated with academic performance, which emphasize the need for preventive and treatment interventions targeted at students’ mental health to improve their psychological well-being and functioning at school.

## Introduction

Psychiatric symptoms, including emotional, social and behavioral problems, may cause functional impairment in multiple areas of life such as school performance and educational attainment^1,2^. For instance, children who exhibit high levels of inattention and hyperactivity have consistently shown to achieve poor grades, low scores on reading and arithmetic standardized tests, and increased rates of grade retention^3^. In addition, significant associations with academic underachievement and failure to complete high school have been found for conduct problems and aggressive behaviors^4–6^. Internalizing forms of psychopathology, on the other hand, may also lead to negative educational outcomes, although mixed results have been reported. Indeed, a meta-analysis based on 26 studies revealed that depression was related to lower school grades, while anxiety showed no significant association^7^. Nevertheless, a controversy remains in the literature since some investigations demonstrated that behavioral and emotional problems did not influence academic performance, when considered together with attention difficulties^8,9^. Thus, not taking into account the substantial co-occurrence of psychiatric symptoms may have biased findings from previous research, which mainly focused on a single aspect of psychopathology. Similarly, when examining the association between mental health problems and academic achievement, it is important to bear in mind that potential common risk factors, such as sociodemographic variables, stressful events and lifestyle behaviors, may cause a spurious effect. In particular, a female advantage in school grades is commonly found, especially for language subjects, and children with healthy lifestyle behaviors, including healthy diets, sufficient sleep and minimal screen time, tend to perform better academically^10–12^. By contrast, low socioeconomic status (SES), ethnicity, and parental separation may increase the likelihood of academic underachievement^13–15^. Finally, grade retention, a standard international practice for dealing with struggling students, have yielded controversial results, with both positive and negative effects on children’s subsequent academic and psychosocial adjustment^16–19^. Indeed, although this corrective measure is supposed to give children a chance to mature and review the educational objectives that were not met during the failed academic year, the preponderance of evidence suggests that academic-related benefits of grade retention, if detected, are limited and tend to diminish over time.

Identifying independent risk factors for academic underachievement is of major importance given that educational failure has negative long-term consequences on health, economic and social outcomes, such as low self-esteem, interpersonal difficulties and antisocial behaviors, lower income, unemployment, and greater use of social welfare^20,21^. However, evidence regarding the effects of mental health problems on school functioning is still inconclusive due to several limitations in previous research. First, many studies used clinically derived cohorts, thus introducing a selection bias and limiting the generalizability of findings. Second, most of the investigations examined relatively small samples and focused on one age group. Third, possible confounding variables such as comorbid psychopathology and environmental conditions have not always been well addressed. Specifically, the joint contribution of children’s lifestyle behaviors to the association between psychiatric symptoms and academic performance represents a gap in the literature since the available reports have failed to account for these factors simultaneously, despite their strong correlation.

Therefore, the present study aims to address these issues by using a large, school-based sample of 7036 students aged 5–17 years. In addition, we examined whether mental health problems were significantly linked to academic achievement, when adjusted for the influence of comorbid psychopathology and a wide range of contextual factors, including sociodemographic variables (i.e., gender, age, educational stage, type of school, SES, ethnicity), stressful life events (i.e., child adoption, parental divorce/separation, grade retention), and lifestyle behaviors (i.e., screen media use, sleep duration, dietary habits).

## Methods

### Participants and procedure

The present study is part of a larger, ongoing research called INSchool aiming to identify children and adolescents’ mental health problems in a school setting, and analyzes data collected over six academic years, from September 2011 through June 2018. The Ministry of Health and the Ministry of Education (Generalitat de Catalunya, Spain) authorized the project, and ethical approval was granted by the Ethics Committee of the Vall d’Hebron Hospital Universitari, in Barcelona, which ensured that all methods were performed in accordance with the relevant guidelines and regulations. A detailed description of the study design, sample, and methodology is provided elsewhere^22^ and briefly summarized here. First, 28 schools from seven different counties in Catalonia were contacted and invited to participate after explaining the procedures to the school staff. All of them accepted, which resulted in 10 418 eligible subjects, with ages comprised between 5 and 17 years (i.e., first grade of primary through fourth grade of secondary education). Informed consent was obtained from 7272 children and their parents/legal guardian (participation rate = 69.8%), 2338 of whom were in secondary education. Parents of the enrolled students completed a questionnaire regarding sociodemographic data, lifestyle and stressful events, along with the Child Behavior Checklist (CBCL)^23^. Information on academic performance was obtained from school records. According to the manual for the Achenbach System of Empirically Based Assessment, individuals with more than eight missing items on the CBCL were removed, which resulted in a final sample of 7036 subjects.

### Measures

#### Psychiatric symptoms

Psychiatric symptoms were assessed with the CBCL^19^, a standardized screening questionnaire that contains a list of 118 emotional and behavioral problems rated by parents from 0 to 2 (0 = *not true*, 1 = *somewhat or sometimes true*, and 2 = *very true or often true*), based on the child’s functioning during the past 6 months. These symptoms are grouped into eight empirically-derived syndrome scales, namely: Withdrawn (e.g., ‘Would rather be alone than with others’, ‘Refuses to talk’, ‘Shy or timid’), Somatic complaints (e.g., ‘Feels dizzy’, ‘Headaches’, ‘Nausea’), Anxious/depressed (e.g., ‘Cries a lot’, ‘Feels he/she has to be perfect’, ‘Nervous, high strung, or tense’), Social problems (e.g., ‘Acts too young for his/her age’, ‘Doesn’t get along with other kids’, ‘Not liked by other kids’), Thought problems (e.g., ‘Repeats certain acts over and over; compulsions’, ‘Strange behavior’, ‘Strange ideas’), Attention problems (e.g., ‘Can’t concentrate, can’t pay attention for long’, ‘Day-dreams or gets lost in his/her thoughts’, ‘Stares blankly’), Delinquent behavior (e.g., ‘Lying or cheating’, ‘Truancy, skips school’, ‘Uses alcohol or drugs for nonmedical purposes’), and Aggressive behavior (e.g., ‘Argues a lot’, ‘Gets in many fights’, ‘Physically attacks people’). Given that norms for Spanish children and adolescents are unavailable, raw scores for each syndrome scale were used as continuous variables to investigate the association between psychopathology and academic performance. Ordinal alpha coefficients yielded adequate reliability estimates across all subscales, with values ranging from 0.83 for Social problems to 0.95 for Aggressive behavior.

#### Potential confounders

##### Sociodemographic factors

Parents completed a questionnaire on sociodemographic data, including child’s gender, age, and country of birth. They also provided information about their educational level, occupation, and country of birth. Students who were not native Spanish or with at least one parent born abroad were considered of foreign origin. Parents’ education and occupation were weighted to compute the Hollingshead four-factor index^24^, a measure of SES ranging from 8 to 66, where higher scores reflect higher SES.

##### Stressful life events

The survey also collected information regarding parental divorce/separation and child adoption. In addition, parents were asked to indicate whether their child had ever repeated a grade.

##### Lifestyle

Parents provided information on their child’s dietary habits by indicating how frequently they had breakfast, lunch, and dinner (1 = *never*, 2 = *occasionally*, and 3 = *always*). They also reported the time the student spent playing videogames, using cellphones, and social networks. Each of these three categories had four possible responses (1 = *never*, 2 = *sometimes*, 3 = *often*, and 4 = *always*) and were summed to compute the mean frequency of screen use. Finally, sleep duration was derived from the answer to the following questions: “At what time does your child usually go to bed?” and “At what time does your child usually wake up?”.

#### Academic performance

Academic performance on four core subjects (i.e., Catalan, Spanish, English as foreign language, and mathematics) was obtained from school records. However, schools varied in the way they scored those subjects and, thus, grades were converted into a 4-point scale from D to A (D = *unsatisfactory achievement*, *fail*, 0–4.9; C = *satisfactory achievement*, *pass*, *average*, 5–6.9; B = *good achievement*, *above average*, 7–8.9; and A = *excellent achievement*, 9–10). Given the high correlation between Catalan and Spanish grades (*r* = 0.76, *p* < 0.001), we calculated the academic performance on first language as the mean of the two scores.

### Statistical analyses

We present descriptive statistics for the sample characteristics, CBCL scores, and grades on first language, foreign language, and mathematics. In order to investigate the independent association between psychopathology and academic performance, four mixed-effects ordinal logistic regression analyses were conducted for each educational outcome (i.e., grades on first language, foreign language, and mathematics) with A as the lowest category, D as the highest, and school as a random effect to take into account the nested structure of data. Multicollinearity diagnostics, based on the variance inflation factor and correlations between independent variables, showed that educational stage was collinear with age. Therefore, we selected age instead of educational stage to be entered in the models. First, we examined mental health problems, sociodemographic factors, stressful life events, and lifestyle variables individually (model 0; crude analyses). In a second set of models, each CBCL syndrome scale was adjusted for gender, age, type of school, SES, and ethnicity (model 1). Model 2 included all dimensions of psychopathology simultaneously, along with sociodemographic factors, to control for co-occurring psychiatric symptoms. Stressful life events and lifestyle variables (i.e., adoption, parental divorce/separation, grade retention, frequency of screen use, sleep duration, and having three meals a day) were added in the fully adjusted models (model 3). The Bonferroni correction for multiple comparisons established the significance threshold at *p* ≤ 0.003, considering the 19 variables assessed. All analyses were performed with SPSS 22.0.

### Missing data

Grades on first language, foreign language, and mathematics were missing for 4.95%, 5.00%, and 4.99% students, respectively. There were also missing values across some other variables. Specifically, the amount of missing data was greatest for the frequency of screen use (9.88%), since this variable was not collected prior to September 2012, followed by sleep duration (7.13%), adoption (5.07%), and parental divorce/separation (4.11%). In contrast, full data were available for gender, age, educational stage, type of school, SES, and CBCL syndrome scores. To preserve sample size and reduce potential missingness bias, missing data were handled using case-wise deletion in all models, which resulted in sample sizes ranging from 4989 to 6602. Children with complete information did not differ in any mental health or academic variable from those who had missing values on sociodemographic factors. However, students with incomplete information on stressful life events and lifestyle reported higher levels of withdrawn (2.82 versus 2.57, *p* < 0.001) and social problems (2.08 versus 1.86, *p* < 0.001), and were more likely to have low grades (first language: odds ratio (*OR*) = 1.34, 95% confidence interval (*CI*) = 1.20–1.50, *p* < 0.001; foreign language: *OR* = 1.25, 95% *CI* = 1.12–1.40, *p* < 0.001; mathematics: *OR* = 1.41, 95% *CI* = 1.26–1.57, *p* < 0.001).

## Results

### Sample characteristics

The sample comprised 3968 (56.4%) boys and 3068 (43.6%) girls with ages ranging from 5 to 17 years (*M* = 9.47; *SD* = 2.91), 68.1% were primary students, and 55.4% attended public schools. The average Hollingshead four-factor index was 43.4 (*SD* = 13.6), which corresponds to a middle-class household income, and subjects had predominantly a Spanish background (82.5%). Those who were not born in Spain came mostly from Spanish-speaking countries (42.9%), Russia (15.9%), China (8.36%), and Morocco (5.29%). In addition, 859 native Spanish children were considered of foreign origin because they had one (57.2%) or both (42.8%) foreign-born parents, the majority of whom came from Latin America and Morocco. One hundred and forty-seven (2.20%) participants were adopted and 19.9% had divorced/separated parents. On average, children were occasionally exposed to screens (*M* = 2.00; *SD* = 0.71) and spent 9.59 h sleeping (*SD* = 0.90; range = 4.75–14.0). Ninety-three percent of them had three meals every day, namely, breakfast, lunch, and dinner.

The mean raw scores for the CBCL syndrome scales were as follows: Withdrawn: 2.66 (*SD* = 2.43; range = 0–18); Somatic complaints: 1.36 (*SD* = 1.75; range = 0–18); Anxious/depressed: 4.54 (*SD* = 4.11; range = 0–27); Social problems: 1.95 (*SD* = 2.18; range = 0–16); Thought problems: 0.49 (*SD* = 1.00; range = 0–14); Attention problems: 4.30 (*SD* = 3.65; range = 0–21); Delinquent behavior: 1.66 (*SD* = 1.87; range = 0–26); and Aggressive behavior: 6.64 (*SD* = 5.67; range = 0–38).

In terms of academic performance, 17.2% students received a grade of A on first language, 37.8% a grade of B, 41.4% a grade of C, and 3.57% a grade of D. Similarly, the proportion of children who achieved a grade of A on foreign language and mathematics was 17.7% and 17.8%, respectively. In contrast, the number of pupils with a grade of D on these subjects was greater (foreign language: 6.64%; mathematics: 8.05%) to the detriment of those with a B (foreign language: 34.0%; mathematics: 34.9%) or a C (foreign language: 41.6%; mathematics: 39.2%). Finally, 5.05% students had repeated a grade at least once.

Table 1 presents the means and standard deviations for the CBCL scales, along with the distribution of sociodemographic, stressful, and lifestyle variables by grade on each academic outcome.

**Table 1:** Distribution of psychiatric symptoms, sociodemographic factors, stressful life events, and lifestyle variables by academic outcomes.

	First language				Foreign language				Mathematics			
	D	C	B	A	D	C	B	A	D	C	B	A
**Psychiatric symptoms (*M*, *SD*)**												
Withdrawn	4.2 (3.3)	3.0 (2.6)	2.3 (2.2)	2.1 (1.9)	4.0 (3.0)	2.9 (2.6)	2.3 (2.1)	2.1 (2.1)	3.6 (3.0)	2.9 (2.6)	2.3 (2.1)	2.2 (2.1)
Somatic complaints	2.1 (2.3)	1.6 (2.0)	1.2 (1.5)	1.1 (1.4)	2.2 (2.4)	1.5 (1.9)	1.2 (1.5)	1.1 (1.5)	2.0 (2.3)	1.5 (1.9)	1.2 (1.5)	1.0 (1.3)
Anxious/depressed	6.4 (5.1)	5.1 (4.3)	4.1 (3.8)	3.6 (3.4)	6.3 (5.1)	4.9 (4.2)	4.0 (3.7)	3.7 (3.6)	5.7 (4.7)	5.0 (4.4)	4.0 (3.7)	3.7 (3.5)
Social problems	3.2 (2.7)	2.4 (2.4)	1.6 (1.9)	1.2 (1.6)	3.1 (2.7)	2.4 (2.3)	1.5 (1.8)	1.2 (1.7)	2.8 (2.6)	2.4 (2.4)	1.6 (1.8)	1.2 (1.6)
Thought problems	1.0 (1.5)	0.6 (1.1)	0.4 (0.9)	0.3 (0.7)	0.9 (1.5)	0.6 (1.1)	0.4 (0.8)	0.3 (0.9)	0.8 (1.2)	0.6 (1.2)	0.4 (0.8)	0.3 (0.8)
Attention problems	7.6 (4.1)	5.5 (3.8)	3.4 (3.1)	2.4 (2.4)	7.2 (4.1)	5.3 (3.8)	3.3 (2.9)	2.6 (2.6)	6.6 (4.1)	5.2 (3.8)	3.4 (3.1)	2.6 (2.5)
Delinquent behavior	3.5 (2.9)	2.0 (1.9)	1.3 (1.6)	1.1 (1.3)	3.1 (2.8)	1.9 (1.9)	1.3 (1.5)	1.1 (1.5)	2.9 (2.7)	1.9 (2.0)	1.3 (1.4)	1.2 (1.4)
Aggressive behavior	10.2 (7.7)	7.7 (5.9)	5.9 (5.2)	4.8 (4.4)	9.8 (7.4)	7.5 (5.8)	5.7 (4.9)	4.9 (4.7)	9.2 (7.0)	7.4 (5.9)	5.8 (4.9)	5.1 (4.7)
**Gender (*n*, %)**												
Boys	175 (73.2)	1725 (62.3)	1302 (51.5)	527 (45.9)	312 (70.3)	1655 (59.5)	1196 (52.6)	562 (47.4)	337 (62.6)	1475 (56.3)	1226 (52.5)	689 (57.9)
Girls	64 (26.8)	1046 (37.7)	1227 (48.5)	622 (54.1)	132 (29.7)	1126 (40.5)	1078 (47.4)	623 (52.6)	201 (37.4)	1147 (43.7)	1108 (47.5)	502 (42.1)
Age (*M*, *SD*)	11.8 (3.1)	9.8 (3.0)	9.1 (2.7)	9.0 (2.7)	12.2 (2.4)	9.5 (3.0)	9.1 (2.7)	9.1 (2.6)	11.8 (2.8)	9.8 (2.9)	8.8 (2. 7)	8.9 (2.6)
**Educational stage (*n*, %)**												
Primary	81 (33.9)	1739 (62.8)	1859 (73.5)	867 (75.5)	117 (26.4)	1832 (65.9)	1694 (74.5)	902 (76.1)	184 (34.2)	1636 (62.4)	1807 (77.4)	915 (76.8)
Secondary	158 (66.1)	1032 (37.2)	670 (26.5)	282 (24.5)	327 (73.6)	949 (34.1)	580 (25.5)	283 (23.9)	354 (65.8)	986 (37.6)	527 (22.6)	276 (23.2)
**Type of school (*n*, %)**												
Public	145 (60.7)	1683 (60.7)	1339 (52.9)	600 (52.2)	258 (58.1)	1671 (60.1)	1210 (53.2)	623 (52.6)	323 (60.0)	1563 (59.6)	1250 (53.6)	625 (52.5)
Private	94 (39.3)	1088 (39.3)	1190 (47.1)	549 (47.8)	186 (41.9)	1110 (39.9)	1064 (46.8)	562 (47.4)	215 (40.0)	1059 (40.4)	1084 (46.4)	566 (47.5)
Socioeconomic status (*M*, *SD*)	33.5 (13.7)	40.1 (14.2)	45.6 (12.4)	48.1 (11.4)	34.4 (13.7)	40.2 (14.1)	45.8 (12.4)	49.2 (10.6)	35.4 (14.3)	40.5 (14.2)	45.6 (12.3)	48.5 (11.0)
**Ethnicity (*n*, %)**												
Native	166 (70.3)	2135 (78.2)	2169 (86.8)	989 (87.0)	318 (72.8)	2181 (79.6)	1954 (86.8)	1000 (85.3)	386 (73.0)	2054 (79.5)	1976 (85.6)	1040 (88.4)
Foreign origin	70 (29.7)	595 (21.8)	330 (13.2)	148 (13.0)	119 (27.2)	558 (20.4)	296 (13.2)	172 (14.7)	143 (27.0)	531 (20.5)	333 (14.4)	136 (11.6)
**Adoption (*n*, %)**												
No	213 (96.8)	2518 (96.8)	2390 (98.6)	1095 (99.1)	385 (96.0)	2551 (97.1)	2147 (98.6)	1131 (99.1)	478 (97.0)	2394 (96.9)	2202 (98.5)	1141 (99.4)
Yes	7 (3.2)	83 (3.2)	34 (1.4)	10 (0.9)	16 (4.0)	75 (2.9)	31 (1.4)	10 (0.9)	15 (3.0)	77 (3.1)	33 (1.5)	7 (0.6)
**Parental divorce/separation (*n*, %)**												
No	144 (62.3)	1970 (74.6)	2050 (84.2)	984 (88.6)	234 (56.0)	2043 (76.9)	1848 (84.4)	1015 (88.3)	310 (60.1)	1926 (77.2)	1907 (84.7)	999 (87.0)
Yes	87 (37.7)	669 (25.4)	385 (15.8)	126 (11.4)	184 (44.0)	612 (23.1)	341 (15.6)	134 (11.7)	206 (39.9)	570 (22.8)	345 (15.3)	149 (13.0)
**Grade retention (*n*, %)**												
No	160 (68.1)	2546 (92.6)	2481 (98.6)	1144 (99.9)	314 (71.2)	2596 (94.3)	2239 (98.9)	1176 (99.4)	409 (76.7)	2442 (94.0)	2292 (98.7)	1183 (99.6)
Yes	75 (31.9)	203 (7.4)	35 (1.4)	1 (0.1)	127 (28.8)	158 (5.7)	24 (1.1)	7 (0.6)	124 (23.3)	157 (6.0)	30 (1.3)	5 (0.4)
Frequency of screen use (*M*, *SD*)	2.5 (0.8)	2.1 (0.7)	1.9 (0.7)	1.8 (0.6)	2.5 (0.7)	2.1 (0.7)	1.9 (0.7)	1.8 (0.6)	2.4 (0.8)	2.1 (0.7)	1.9 (0.6)	1.9 (0.6)
Sleeping hours (*M*, *SD*)	9.1 (1.1)	9.5 (0.9)	9.7 (0.9)	9.7 (0.8)	9.00 (1.0)	9.6 (0.9)	9.7 (0.8)	9.7 (0.8)	9.1 (1.1)	9.5 (0.9)	9.7 (0.8)	9.7 (0.8)
**Three meals a day (*n*, %)**												
No	41 (17.4)	261 (9.5)	115 (4.6)	34 (3.0)	76 (17.2)	235 (8.5)	98 (4.3)	43 (3.7)	93 (17.4)	229 (8.8)	88 (3.8)	41 (3.5)
Yes	195 (82.6)	2493 (90.5)	2405 (95.4)	1108 (97.0)	365 (82.8)	2532 (91.5)	2164 (95.7)	1135 (96.3)	440 (82.6)	2376 (91.2)	2240 (96.2)	1142 (96.5)

Differences in sample sizes across variables are due to missing data.

### Association analyses with academic performance

Unadjusted and adjusted *OR* from the mixed-effects ordinal logistic regression models are reported in Tables 2, 3 and 4. Crude analyses revealed that higher scores on the CBCL syndrome scales were significantly associated with lower grades on first language, foreign language, and mathematics (*p* < 0.001; Tables 2, 3, 4, model 0). Risk for academic underachievement also increased with age and among children of foreign origin, while students from higher SES were less likely to have low grades (*p* < 0.001; Tables 2, 3, 4, model 0). Gender was only related to performance on language subjects, where girls achieved better grades than boys (first language: *OR* = 0.55, 95% *CI* = 0.50–0.60, *p* < 0.001; foreign language: *OR* = 0.58, 95% *CI* = 0.53–0.64, *p* < 0.001), and no significant differences were found between pupils attending public and private institutions once the random effect of school was controlled for. With regard to stressful life events, the strongest association was observed for repeater students, followed by adopted children, or with divorced/separated parents (*p* < 0.001; Tables 2, 3, 4, model 0). Finally, participants who had three meals a day and spent more time sleeping exhibited higher academic performance. In contrast, those more frequently exposed to screens were at risk for underachievement (*p* < 0.001; Tables 2, 3, 4, model 0).

**Table 2:** Mixed-effects ordinal logistic regression models of poor academic performance on first language.

Independent variables	Model 0	Adjusted *OR* (95% *CI*)		
		Model 1	Model 2	Model 3
**Psychiatric symptoms**				
Withdrawn	1.16 (1.13–1.18)*	1.12 (1.10–1.14)*	1.03 (1.00–1.05)	1.02 (0.99–1.05)
Somatic complaints	1.14 (1.11–1.18)*	1.11 (1.08–1.15)*	1.00 (0.97–1.03)	0.99 (0.95–1.03)
Anxious/depressed	1.08 (1.07–1.09)*	1.06 (1.05–1.08)*	0.94 (0.92–0.95)*	0.93 (0.91–0.95)*
Social problems	1.26 (1.23–1.29)*	1.22 (1.19–1.25)*	1.02 (0.99–1.06)	1.02 (0.98–1.06)
Thought problems	1.37 (1.30–1.44)*	1.29 (1.22–1.35)*	0.84 (0.78–0.89)*	0.82 (0.77–0.89)*
Attention problems	1.25 (1.23–1.27)*	1.23 (1.21–1.24)*	1.28 (1.25–1.31)*	1.26 (1.23–1.29)*
Delinquent behavior	1.31 (1.28–1.35)*	1.25 (1.22–1.29)*	1.09 (1.05–1.13)*	1.06 (1.01–1.10)
Aggressive behavior	1.09 (1.08–1.10)*	1.08 (1.07–1.09)*	0.99 (0.98–1.01)	1.00 (0.98–1.02)
**Sociodemographic factors**				
Gender (ref. Boys)	0.55 (0.50–0.60)*		0.73 (0.66–0.81)*	0.67 (0.60–0.76)*
Age	1.12 (1.09–1.14)*		1.10 (1.08–1.13)*	1.07 (1.03–1.10)*
Type of school (ref. Public)	0.68 (0.41–1.15)		1.09 (0.71–1.66)	1.17 (0.78–1.75)
Socioeconomic status	0.96 (0.95–0.96)*		0.96 (0.96–0.97)*	0.96 (0.96–0.97)*
Ethnicity (ref. Native)	1.67 (1.47–1.90)*		1.35 (1.18–1.54)*	1.16 (0.99–1.36)
**Stressful life events**				
Adoption (ref. No)	2.61 (1.86–3.66)*			1.92 (1.25–2.96)
Parental divorce/separation (ref. No)	1.96 (1.74–2.22)*			1.36 (1.17–1.58)*
Grade retention (ref. No)	9.47 (7.32–12.2)*			3.38 (2.45–4.65)*
**Lifestyle variables**				
Frequency of screen use	1.51 (1.40–1.63)*			1.09 (0.99–1.20)
Sleeping hours	0.85 (0.79–0.90)*			1.01 (0.92–1.11)
Three meals a day (ref. No)	0.45 (0.37–0.55)*			0.68 (0.53–0.86)*

Model 0: crude analysis; Model 1 (*n* = 6602): adjusted for sociodemographic factors; Model 2 (*n* = 6602): adjusted for sociodemographic factors and other mental health problems; Model 3 (*n* = 4992): adjusted for sociodemographic factors, other mental health problems, stressful life events, and lifestyle variables.

*OR* odds ratio, *CI* confidence interval.

*Significance threshold for the Bonferroni correction at *p* ≤ 0.003.

**﻿Table 3:** Mixed-effects ordinal logistic regression models of poor academic performance on foreign language.

Independent variables	Model 0	Adjusted *OR* (95% *CI*)		
		Model 1	Model 2	Model 3
**Psychiatric symptoms**				
Withdrawn	1.16 (1.13–1.18)*	1.13 (1.10–1.15)*	1.04 (1.02–1.07)*	1.03 (1.00–1.06)
Somatic complaints	1.14 (1.11–1.17)*	1.11 (1.08–1.14)*	1.00 (0.97–1.03)	1.00 (0.97–1.04)
Anxious/depressed	1.07 (1.06–1.09)*	1.06 (1.05–1.07)*	0.93 (0.91–0.95)*	0.93 (0.91–0.95)*
Social problems	1.25 (1.22–1.28)*	1.21 (1.18–1.24)*	1.03 (1.00–1.06)	1.03 (0.99–1.07)
Thought problems	1.34 (1.28–1.40)*	1.28 (1.21–1.34)*	0.85 (0.80–0.91)*	0.84 (0.78–0.90)*
Attention problems	1.23 (1.21–1.24)*	1.20 (1.19–1.22)*	1.25 (1.22–1.28)*	1.23 (1.20–1.26)*
Delinquent behavior	1.30 (1.27–1.33)*	1.24 (1.20–1.27)*	1.08 (1.04–1.12)*	1.03 (0.99–1.08)
Aggressive behavior	1.09 (1.08–1.09)*	1.07 (1.07–1.08)*	1.00 (0.99–1.01)	1.00 (0.99–1.02)
**Sociodemographic factors**				
Gender (ref. Boys)	0.58 (0.53–0.64)*		0.78 (0.70–0.86)*	0.77 (0.68–0.87)*
Age	1.12 (1.09–1.14)*		1.12 (1.09–1.14)*	1.07 (1.04–1.10)*
Type of school (ref. Public)	0.76 (0.41–1.38)		1.29 (0.86–1.93)	1.27 (0.87–1.87)
Socioeconomic status	0.95 (0.95–0.96)*		0.96 (0.95–0.96)*	0.96 (0.96–0.97)*
Ethnicity (ref. Native)	1.50 (1.32–1.70)*		1.16 (1.02–1.32)	0.93 (0.79–1.08)
**Stressful life events**				
Adoption (ref. No)	2.71 (1.94–3.78)*			2.16 (1.41–3.29)*
Parental divorce/separation (ref. No)	2.04 (1.81–2.30)*			1.41 (1.22–1.64)*
Grade retention (ref. No)	9.80 (7.71–12.5)*			3.84 (2.83–5.21)*
**Lifestyle variables**				
Frequency of screen use	1.50 (1.39–1.62)*			1.09 (0.99–1.20)
Sleeping hours	0.81 (0.75–0.86)*			0.96 (0.87–1.05)
Three meals a day (ref. No)	0.48 (0.40–0.58)*			0.74 (0.59–0.93)

Model 0: crude analysis; Model 1 (*n* = 6598): adjusted for sociodemographic factors; Model 2 (*n* = 6598): adjusted for sociodemographic factors and other mental health problems; Model 3 (*n* = 4989): adjusted for sociodemographic factors, other mental health problems, stressful life events, and lifestyle variables.

*OR* odds ratio, *CI* confidence interval.

*Significance threshold for the Bonferroni correction at *p* ≤ 0.003.

**Table 4:** Mixed-effects ordinal logistic regression models of poor academic performance on mathematics.

Independent variables	Model 0	Adjusted *OR* (95% *CI*)		
		Model 1	Model 2	Model 3
**Psychiatric symptoms**				
Withdrawn	1.12 (1.10–1.14)*	1.09 (1.07–1.11)*	0.99 (0.96–1.02)	0.98 (0.95–1.01)
Somatic complaints	1.15 (1.12–1.18)*	1.11 (1.08–1.14)*	1.01 (0.98–1.05)	1.01 (0.97–1.04)
Anxious/depressed	1.07 (1.06–1.08)*	1.06 (1.04–1.07)*	0.95 (0.93–0.97)*	0.95 (0.93–0.97)*
Social problems	1.23 (1.20–1.26)*	1.19 (1.17–1.22)*	1.03 (1.00–1.07)	1.03 (1.00–1.07)
Thought problems	1.31 (1.25–1.37)*	1.25 (1.19–1.31)*	0.87 (0.82–0.93)*	0.85 (0.79–0.91)*
Attention problems	1.20 (1.18–1.22)*	1.19 (1.17–1.21)*	1.23 (1.20–1.26)*	1.22 (1.19–1.25)*
Delinquent behavior	1.28 (1.24–1.31)*	1.24 (1.20–1.27)*	1.11 (1.07–1.15)*	1.08 (1.04–1.13)*
Aggressive behavior	1.07 (1.07–1.08)*	1.07 (1.06–1.08)*	0.99 (0.98–1.00)	1.00 (0.98–1.01)
**Sociodemographic factors**				
Gender (ref. Boys)	0.93 (0.85–1.02)		1.29 (1.17–1.43)*	1.23 (1.09–1.38)*
Age	1.14 (1.12–1.16)*		1.13 (1.11–1.16)*	1.10 (1.06–1.13)*
Type of school (ref. Public)	0.68 (0.36–1.28)		1.05 (0.70–1.57)	1.18 (0.85–1.64)
Socioeconomic status	0.96 (0.95–0.96)*		0.97 (0.96–0.97)*	0.97 (0.96–0.97)*
Ethnicity (ref. Native)	1.66 (1.46–1.88)*		1.34 (1.18–1.53)*	1.19 (1.02–1.39)
**Stressful life events**				
Adoption (ref. No)	2.59 (1.86–3.60)*			1.81 (1.19–2.74)
Parental divorce/separation (ref. No)	1.91 (1.70–2.15)*			1.29 (1.11–1.49)*
Grade retention (ref. No)	6.87 (5.44–8.67)*			2.96 (2.20–3.97)*
**Lifestyle variables**				
Frequency of screen use	1.49 (1.38–1.61)*			1.08 (0.98–1.19)
Sleeping hours	0.82 (0.77–0.87)*			1.03 (0.94–1.13)
Three meals a day (ref. No)	0.43 (0.36–0.52)*			0.73 (0.58–0.92)

Model 0: crude analysis; Model 1 (*n* = 6599): adjusted for sociodemographic factors; Model 2 (*n* = 6599): adjusted for sociodemographic factors and other mental health problems; Model 3 (*n* = 4991): adjusted for sociodemographic factors, other mental health problems, stressful life events, and lifestyle variables.

*OR* odds ratio, *CI* confidence interval.

*Significance threshold for the Bonferroni correction at *p* ≤ 0.003.

The association between mental health problems and poor academic achievement was sustained after adjustment for sociodemographic factors, although the *OR* were slightly reduced (*p* < 0.001; Tables 2, 3, 4, model 1). Nevertheless, when we additionally included all the CBCL scales in the model to account for comorbid psychopathology, somatic complaints, social problems, and aggressive behavior were no longer related to educational outcomes (Tables 2, 3, 4, model 2). The effect of anxious/depressed symptoms, thought problems, attention problems, and delinquent behavior, on the other hand, remained significant. Specifically, students who reported more anxious/depressed and thought problems were less likely to perform poorly, while those with increased levels of attention problems and delinquent behavior had higher risk for academic impairment (*p* < 0.001; Tables 2, 3, 4, model 2). Finally, scores on the Withdrawn scale were only associated with lower grades on foreign language (*OR* = 1.04, 95% *CI* = 1.02–1.07, *p* = 0.002).

**Figure 1:**
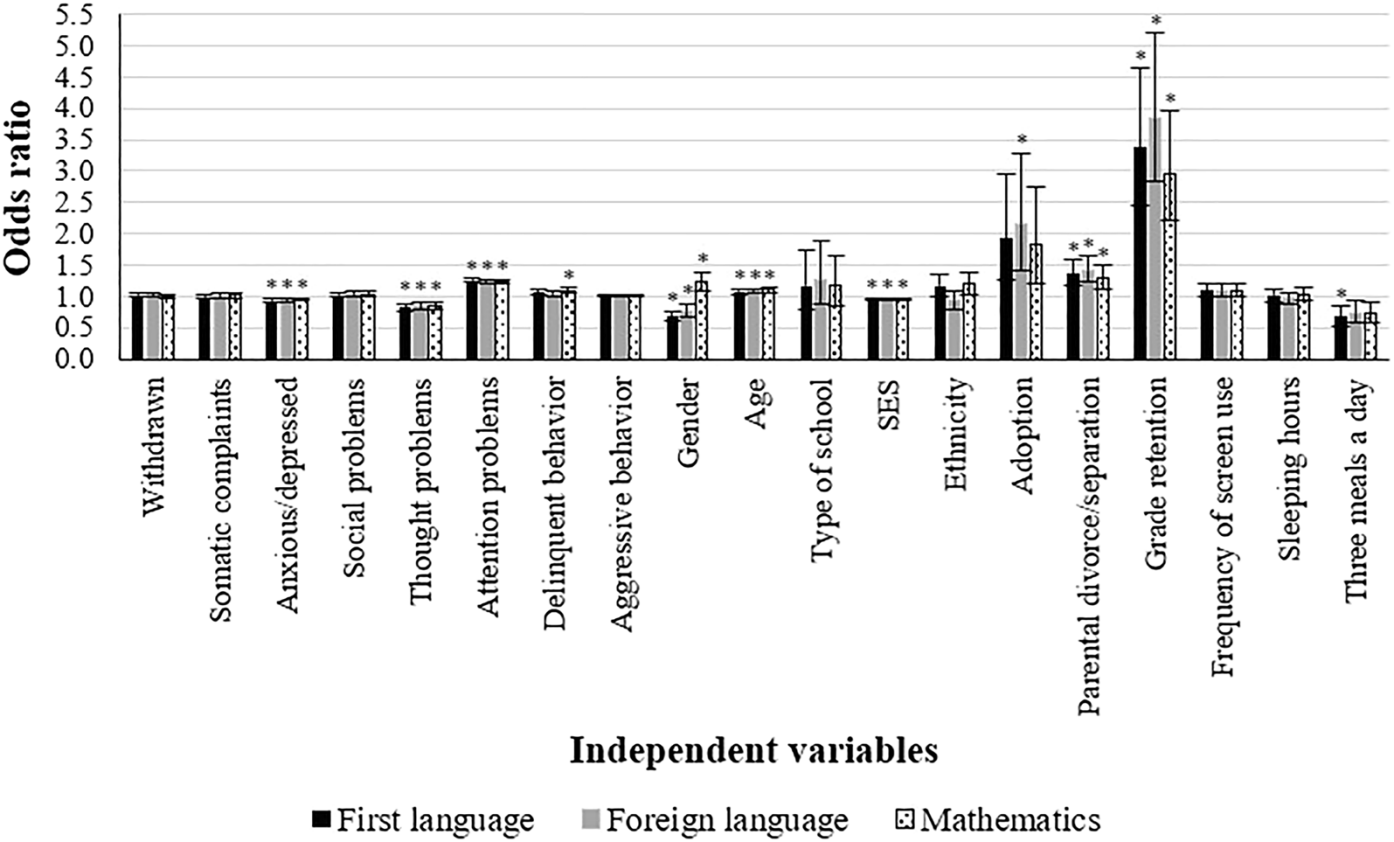
Odds ratios and 95% confidence intervals from the fully adjusted models for poor academic performance on each of the educational outcomes. *Significance threshold for the Bonferroni correction at *p* ≤ 0.003.

In the fully adjusted models, the *OR* for the anxious/depressed, thought, and attention problems were mainly the same (*p* < 0.001; Tables 2, 3, 4, model 3). Once stressful life events and lifestyle variables were controlled for, however, the relationship between delinquent behavior and poor academic performance attenuated to a non-significant level, except for mathematics (*OR* = 1.08, 95% *CI* = 1.04–1.13, *p* < 0.001). Besides, ethnicity, frequency of screen use, and sleep duration were no longer associated with educational outcomes when the influence of psychopathology and all potential confounders was taken into account. Similarly, the effect of having three meals a day and adoption declined and was only sustained for grades on first language and foreign language, respectively (*OR* = 0.68, 95% *CI* = 0.53–0.86, *p* = 0.001 and *OR* = 2.16, 95% *CI* = 1.41–3.29, *p* < 0.001). The remaining variables were still related to academic outcomes in model 3 (Tables 2, 3, 4) and gender also became significant for mathematics, where girls performed worse than boys (*OR* = 1.23, 95% *CI* = 1.09–1.38, *p* = 0.001). Figure 1 shows the *OR* and 95% *CI* from the fully adjusted models for each of the educational outcomes.

## Discussion

The present study aimed to complement and expand previous research on the relationship between mental health problems and academic performance by examining simultaneously multiple dimensions of psychopathology (i.e., withdrawn, somatic complaints, anxious/depressed symptoms, social problems, thought problems, attention problems, delinquent behavior, and aggressive behavior) and environmental conditions (i.e., sociodemographic variables, stressful life events, and lifestyle behaviors) in a school-based sample of 7036 Spanish children and adolescents. Taken together, our findings highlight the relevance of anxious/depressed symptoms, thought problems, attention problems, and delinquent behavior for academic achievement, even when symptoms are not extreme enough to attract a diagnosis and regardless of comorbid psychopathology and other risk factors.

First, when psychiatric symptoms were examined separately, we found that higher scores on the CBCL syndrome scales were significantly associated with lower grades, even after controlling for sociodemographic variables. These results add to a wealth of data demonstrating a negative impact of depressive symptoms, anxiety, attention and conduct problems on academic outcomes^2,8,25,26^. However, correlations among emotional and behavioral assessments must be taken into account, as suggested by earlier reports from North America, which showed that, when considered together, only attention problems predicted later academic achievement, while the influence of externalizing and internalizing psychopathology was no longer significant^8,9,27^. In contrast, longitudinal studies conducted in Europe provide evidence for a link between externalizing problems and educational attainment, after adjustment for co-occurring symptoms, background factors, and school difficulties prior to the baseline^28,29^. Specifically, Galéra et al.^28^ revealed that both conduct disorder and hyperactivity-inattention symptoms contributed independently to heighten the risk of grade retention, failure in secondary graduation examination, lower diploma achievement, and poorer academic performance in a large French population-based sample. Likewise, Sagatun et al.^29^ reported an association between externalizing problems and non-completion of upper secondary school during the following 5 years, but also found that girls who did not complete school exhibited more internalizing symptoms in the 10th grade, regardless of other risk factors. Consistent with this last survey from Norway, our findings indicated an effect of anxious/depressed symptoms, thought problems, attention problems, and delinquent behavior on educational outcomes, when we additionally included all the CBCL scales in the model to control for comorbid psychopathology. According to previous studies, which point to inattention as the strongest factor associated with academic underachievement^8,30–33^, students with increased levels of attention problems were the most likely to perform poorly on all academic subjects. These children are easily distracted, often appears not to be listening, and have difficulties concentrating on one task, holding attention for long, following instructions, or completing school work^2,28,34^. Thus, it is unsurprising that they experience problems in an academic environment, which may lead to lower levels of achievement. Besides, our data support the hypothesis that conduct disorder symptoms may undermine academic performance beyond its correlation with attention problems, since delinquent behavior independently accounted for the risk of lower grades. In this sense, it has been proposed that delinquent behavior, referred to the involvement in illegal activities, substance use, school misbehavior and truancy, may affect students’ engagement with schooling and influence their grades through negative feedback and repeated disciplinary actions, such as suspension or expulsion^35^. Nevertheless, we cannot rule out alternative explanations, especially considering that the relationship between delinquent behavior and poor academic performance only remained significant for mathematics once stressful life events were taken into account. Strikingly, on the other hand, individuals who reported more anxious/depressed problems achieved better grades. Although somewhat counterintuitive, this result is in line with other investigations indicating a positive relationship between anxiety symptoms and academic performance^32,36–38^. Of note, Fernández-Castillo and Gutiérrez-Rojas^36^ examined the presence of emotional disturbance, depression, and selective attention deficit in 98 students enrolled in two secondary education schools from the city of Granada, Spain, and found that moderate levels of anxiety were associated with improved academic achievement when the influence of depression and attention were controlled. Similarly, Voltas et al.^37^ showed that higher generalized anxiety and social phobia symptoms predicted greater academic performance by using a three-phase prospective study with an initial sample of 1514 Spanish scholars aged 9–12 years. There may be several explanations for this effect. On one hand, moderate levels of anxiety produce a state of alert and tension that may lead to better achievement in tasks that require attention^36^. On the other, anxiety has been related to conscientiousness, discipline, and perfectionism^39^. As a result, the motivation to work hard and perform well may be higher in subjects exhibiting anxiety symptoms to meet the school’s requirements and avoid failure or negative evaluations^40^. In this vein, perfectionism might also explain that students who reported more thought problems were less likely to perform poorly, since some of its behavioral components, such as careful checking, reassurance seeking, and excessive consideration before making a decision, may be motivated by the fear of failure and have been linked to symptoms of obsessive–compulsive disorder^40^. Interestingly, a prospective cohort study of Dutch preadolescents had previously found that teachers’ reports of thought problems were associated with better academic performance in boys at secondary school^32^. Overall, our results suggest that anxiety may enhance academic achievement, as long as symptoms are not clinically relevant and pupils have resources to manage their motivation properly and deal with aversive emotions. Nevertheless, in order to draw clear conclusions, future studies should include specific instruments that investigate the effects of depression and anxiety separately, as they may have different relationships with subsequent school attainment^7^.

With regard to sociodemographic variables, our findings corroborate the positive relationship between SES and academic achievement observed in previous national and international investigations^14,37,41–43^. The higher performance of girls on language subjects was not surprising either, according to a meta-analysis which demonstrated the presence of a stable female advantage in school marks, especially for language courses^15^. On the contrary, we found that girls achieved lower grades than boys on mathematics in the fully adjusted model. Likewise, a systematic review of the Spanish results from the OECD’s Programme for International Student Assessment (PISA) revealed notable gender differences depending on the area of knowledge and skills assessed, with girls scoring higher on reading while boys performed better in mathematics and science^42^. Furthermore, the increased risk for academic underachievement with age echoes findings of a large Dutch longitudinal cohort study, where school functioning tended to worsen from primary to secondary education^32^, probably because of the hormonal changes, higher social expectations, and more challenging academic environment during adolescence (e.g., more teachers, different classes, greater academic demands)^44^. We also observed that repeater students were approximately three times more likely to display negative academic outcomes than their classmates, thus suggesting that grade retention might be less helpful for struggling children than generally thought. In this vein, previous findings, including those from the PISA study in Spain, have generally fail to demonstrate that repeating a grade provides many advantages in terms of academic performance, psychosocial adjustment, and school career^17,18,42^. Hence, the present investigation fuels the ongoing pedagogical debate about whether this practice really grants pupils with difficulties more time to mature, catch up, and acquire the basic knowledge and skills necessary for promoting to the next grade level, where more advanced learning tasks have to be dealt with.

Another controversial issue arising from our research concerns the effects of students’ health behaviors, namely diet, sleep and screen time, on their education. Specifically, academic achievement has been negatively related to excessive screen time and inappropriate sleep duration^11,45^. Nevertheless, recent reviews noted that studies have rarely examined these behaviors simultaneously or considered other potential mediators and moderators^46^. Indeed, we found that participants who spent more time sleeping exhibited higher academic performance, while those who were more frequently exposed to screens had higher risk for underachievement, although these associations attenuated to a non-significant level when the influence of psychopathology, sociodemographic factors, stressful events, and lifestyle variables was taken into account. Interestingly, excessive smartphone use has been linked to several impulsivity facets, increased levels of general anxiety and depressive symptoms, low self-esteem, and high neuroticism^47,48^. In this sense, it has been hypothesized that individuals with poor self-control might be at risk for developing problematic mobile phone use. On the other hand, subjects characterized by low levels of self-esteem and high neuroticism might use the cellphone excessively to obtain a constant reassurance in affective relationships promoted by increased emotional instability, insecure attachment, and the fear of being rejected^47^. Moreover, the use of screen-based activities in the evening often delays bedtime, which compromises sleep duration and quality^49^. Lastly, recent studies have also related some of the aforementioned personality traits, namely neuroticism and impulsivity, to insufficient sleep. For instance, a longitudinal investigation found that adolescents scoring high on impulsive urgency were particularly prone to report symptoms of insomnia, hyperactivity and poor school grades, with hyperactivity partially mediating the negative effects of sleep problems on school performance^50^.

The current research, however, should be interpreted in light of several limitations. The cross-sectional design of the study hinders the ability to establish conclusions on causal relationships and, thus, we cannot rule out that psychopathology could have resulted from negative academic outcomes. Although differences between the complete sample and subjects with missing data on stressful life events and lifestyle were small, it is unknown to what extent the loss of these participants might have impacted the findings. Indeed, associations might have actually been attenuated in our study, as they had higher levels of withdrawn and social problems, and poorer academic performance than included children. Finally, alternative explanations for the observed relationship between mental health problems and academic performance cannot be excluded either, since other variables, such as children’s intelligence quotient, executive functioning or treatment status, which might play a confounding role, were not considered. By contrast, we adjusted our analyses for multiple environmental risk factors, co-occurring psychiatric symptoms, the nested structure of data within schools, and the number of predictors added in the final model. Additional strengths include the large size and age range of the sample, composed of 7036 children and youth aged 5–17 years, and the use of real-life measures of academic performance in a school-based rather than a clinical sample, which produces a more valid reflection of the general population. Furthermore, by using different informants for children’s mental health problems and academic performance we were able to limit shared method variance, which occurs when the same source is used to report on both the determinant and the outcome^51^. For instance, teachers are likely the best informants with regard to academic functioning in the class, but relying on them to also assess child psychopathology might have strongly inflate associations since they tend to rate problem behavior of children with low grades more negatively than parents^1^. In this sense, parents are found to be good identifiers of both externalizing and internalizing symptoms, especially for children and young adolescents, who might be less able to judge and rate their own behavior^52,53^. Older youth, on the other hand, may be better at reporting emotional difficulties as they have privileged access to their inner distress, although self-reports are more prone to distortions arising from social desirability^53,54^. Hence, given that each informant presents a different perspective, future studies should attempt to combine multiple reports on psychopathology to increase the accuracy of the measurement.

Overall, the present investigation demonstrates that anxious/depressed symptoms and thought problems are positively related to academic achievement, while students with attention problems and delinquent behavior are more likely to perform poorly, even when symptoms are not extreme enough to attract a diagnosis and regardless of other risk factors, such as comorbid psychopathology, sociodemographic variables, stressful events, and lifestyle behaviors. This emphasizes the need for assessment and treatment strategies targeted at students’ mental health in order to improve their psychological well-being and functioning at school. In this vein, a Canadian study found that early preventive intervention for individuals at risk of antisocial behavior increased the rates of high-school completion while, in Chile, the world’s largest school-based mental health program led to better academic progress among students whose mental health problems improved^55,56^. Additional programs should provide children who do not struggle in school but experience aversive emotions with resources to manage their motivation properly and cope with stress. Finally, in order to account for co-occurring symptoms, future studies on psychopathology and academic achievement should take advantage from person-centered approaches, such as latent profile analysis. This analytic strategy could be used to form subgroups of students based on their symptom presentations and determine whether the emergent profiles are differentially associated with academic performance. Research using longitudinal data to identify the causal relationship between psychopathology and educational outcomes would also be beneficial.

## Data Availability

The datasets generated and/or analyzed during the current study are available from the corresponding author on reasonable request.
